# Limited Restoration of Contrast Sensitivity with Training after V1 Damage in Humans

**DOI:** 10.1523/ENEURO.0020-24.2024

**Published:** 2024-03-12

**Authors:** Jingyi Yang (杨菁艺), Elizabeth L. Saionz, Matthew R. Cavanaugh, Berkeley K. Fahrenthold, Michael D. Melnick, Duje Tadin, Farran Briggs, Marisa Carrasco, Krystel R. Huxlin

**Affiliations:** ^1^Flaum Eye Institute, University of Rochester Medical Center, Rochester, New York 14642; ^2^Department of Neuroscience, University of Rochester Medical Center, Rochester, New York 14642; ^3^Center for Visual Science, University of Rochester, Rochester, New York 14627; ^4^Department of Brain & Cognitive Sciences, University of Rochester, Rochester, New York 14627; ^5^Department of Psychology and Center for Neural Science, New York University, New York, New York 10003

**Keywords:** blindness, direction, discrimination, hemianopia, orientation, perceptual learning

## Abstract

Stroke damage to the primary visual cortex (V1) causes severe visual deficits, which benefit from perceptual retraining. However, whereas training with high-contrast stimuli can locally restore orientation and motion direction discrimination abilities at trained locations, it only partially restores luminance contrast sensitivity (CS). Recent work revealed that high-contrast discrimination abilities may be preserved in the blind field of some patients early after stroke. Here, we asked if CS for orientation and direction discrimination is similarly preserved inside the blind field, to what extent, and whether it could benefit from a visual training intervention. Thirteen subacute patients (<3 months post-V1 stroke) and 12 chronic patients (>6 months post-V1 stroke) were pretested and then trained to discriminate either orientation or motion direction of Gabor patches of progressively lower contrasts as their performance improved. At baseline, more subacute than chronic participants could correctly discriminate the orientation of high-contrast Gabors in their blind field, but all failed to perform this task at lower contrasts, even when 10 Hz flicker or motion direction were added. Training improved CS in a greater portion of subacute than that of chronic participants, but no one attained normal CS, even when stimuli contained flicker or motion. We conclude that, unlike the near-complete training-induced restoration of high-contrast visual discrimination abilities, V1 damage in adulthood may severely limit the residual visual system's ability to regain normal CS. Our results support the notion that CS involves different neural substrates and computations than those required for orientation and direction discrimination in V1-damaged visual systems.

## Significance Statement

Stroke-induced V1 damage in adult humans induces a rapid and severe impairment of contrast sensitivity (CS) for orientation and motion direction discrimination in the affected hemifield, although discrimination of high-contrast stimuli can persist for several months. Adaptive training with Gabor patches of progressively lower contrasts improves CS for both orientation and motion discriminations in the blind field of subacute (<3 months poststroke) and chronic (>6 months poststroke) participants; however, it fails to restore normal CS. Nonetheless, more subacute than chronic stroke participants benefit from such training, particularly when discriminating the orientation of static, nonflickering targets. Thus, CS appears critically dependent on processing within V1, with perceptual training displaying limited potential to fully restore it after V1 damage.

## Introduction

Damage to the primary visual cortex (V1) causes cortically induced blindness (CB)—a homonymous visual impairment affecting both eyes, which afflicts 25–50% of stroke patients (Zhang et al., 2006; Hazelton et al., 2019). By 6 months poststroke, CB participants are considered “chronic” and exhibit pronounced defects in detecting and discriminating targets in the hemifield contralateral to their V1 damage (reviewed by Saionz et al., 2022). Indeed, most CB patients fail to consciously discriminate opposite motion directions or differences in orientation (Das et al., 2014; Cavanaugh et al., 2015, 2019, 2023) and they cannot effectively integrate across motion directions (Huxlin et al., 2009; Das et al., 2014; Cavanaugh and Huxlin, 2017; Saionz et al., 2020) in their blind field. In addition, spatial and temporal contrast sensitivity (CS) functions for discrimination can no longer be reliably measured inside perimetrically defined blind fields (Hess and Pointer, 1989; Clatworthy et al., 2013; Das et al., 2014). As an essential attribute of vision (Kelly, 1975; Campbell, 1983), the restoration of CS is thus highly sought-after in visually impaired populations.

Visual training protocols—usually administered with high-contrast stimuli—are increasingly used in attempts to restore visual abilities inside CB fields. Functions that can be improved with such training include fine and coarse direction discrimination and integration (Huxlin et al., 2009; Das et al., 2014; Cavanaugh et al., 2015, 2019; Elshout et al., 2016), simple orientation and direction discrimination (Das et al., 2014), detection of flickering gratings (Sahraie et al., 2006, 2010; Trevethan et al., 2012), relative target localization (Chokron et al., 2008; Elshout et al., 2016), flicker sensitivity (Raninen et al., 2007), motion coherence (Vaina et al., 2014), and letter identification (Raninen et al., 2007; Chokron et al., 2008). Some of these training interventions reduced the size of perimetrically defined blind fields (Elshout et al., 2016; Cavanaugh and Huxlin, 2017) and improved luminance CS, although in no case did CS return to normal (Huxlin et al., 2009; Das et al., 2014; Saionz et al., 2020). However, restoring normal CS was not the intended goal in these prior studies, and with a few exceptions involving detection training (Sahraie et al., 2006, 2010; Trevethan et al., 2012), they all used high-contrast stimuli. This reliance on highly visible stimuli makes sense for vision restoration in CB patients, who tend to have flat contrast sensitivity functions (CSFs) in their blind field, at baseline (Huxlin et al., 2009; Das et al., 2014; Saionz et al., 2020). However, never exposing or requiring the visual system to discriminate low-contrast stimuli may also be a poor strategy to restore sensitivity to such stimuli; instead, training that requires the visual system to detect or discriminate targets of increasingly low contrast have shown efficacy in conditions such as amblyopia (Zhou et al., 2006) and improved CS in visually intact controls (Xi et al., 2020).

An inability to recover normal CS at trained, blind-field locations means that restored vision in CB remains fundamentally degraded and less useful in everyday life. Here, we considered three possible reasons as to why CS fails to fully recover at trained, blind-field locations in V1-damaged participants: (1) training with only high-contrast stimuli, as in most CB vision restoration studies, might not be optimal for improving CS; (2) CS recovery may depend on when training is administered poststroke (Saionz et al., 2020); and (3) residual visual circuitry is permanently altered by the V1 damage. In intact visual systems, CS correlates positively with V1 surface area (Himmelberg et al., 2022). V1 neurons are highly selective to contrast and underlie contrast discrimination (Albrecht and Hamilton, 1982; Boynton et al., 1999). If these neurons are lost, the residual visual system may simply lack the neural circuitry necessary to fully recover CS, regardless of timing of training and/or training protocol. To arbitrate among these possibilities, we compared the efficacy of training in subacute versus chronic CB stroke patients with stimuli of progressively lower luminance contrasts, at spatial (SFs) and temporal frequencies (TFs) optimal for stimulus detection and discrimination in the blind field (Sahraie et al., 2003, 2008). We show that perceptual training targeted at the deficient function (discriminating low-contrast targets), bootstrapped to tasks (orientation and direction discrimination) known to elicit improvements in this patient population (Huxlin et al., 2009; Das et al., 2014; Saionz et al., 2020), fails to restore normal CS in both subacute and chronic patients with V1 damage.

## Materials and Methods

### Participants

**Figure 1.**
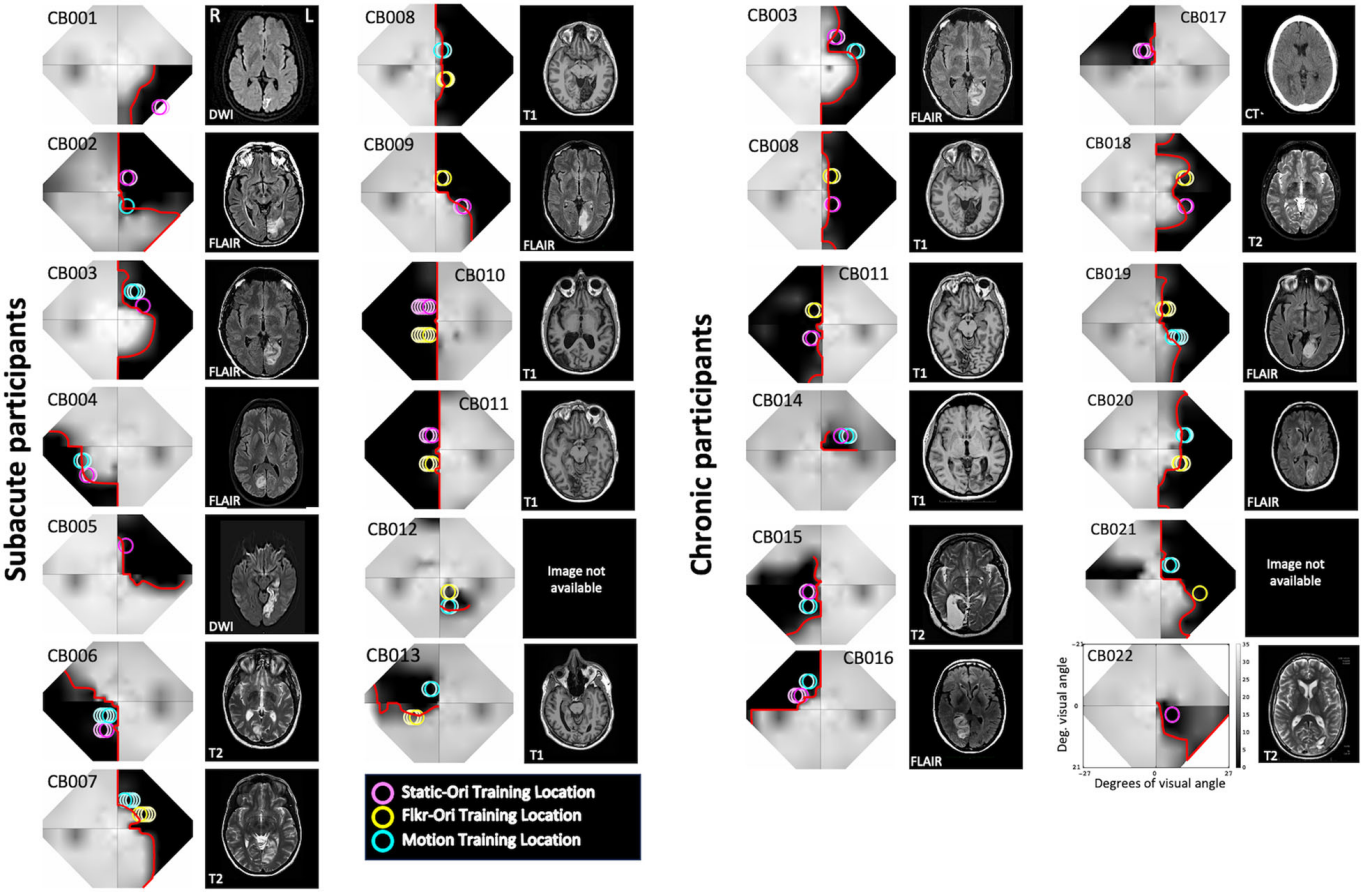
Composite visual field maps, locations trained, and brain scans for individual CB participants separated into subacute and chronic groups. Humphrey perimetry-derived, binocular maps of visual sensitivity (gray scale next to CB022, dB) across the central visual field, alongside sample clinical brain images illustrating stroke damage to the occipital cortex. Image type [T1, T2, diffusion-weighted imaging (DWI), T2-weighted fluid-attenuated inversion recovery (FLAIR) for magnetic resonance imaging; computed tomography (CT)] are indicated on each radiographic image, shown according to radiographic convention with left brain hemisphere (L) on the right image. In visual field maps, the red line denotes the blind-field border encircling regions of mean Humphrey sensitivity <10 dB, the Social Security Administration definition of blindness (see main text for details). Colored circles on each visual field map represent the locations and size of Gabor stimuli used to train participants on the three different discrimination tasks, Static-Ori (Task 1), Flkr-Ori (Task 2), and Motion (Task 3), with the brightest circles denoting the locations initially selected for training, whose performance was verified in-lab pre- and post-training, and whose data are analyzed in Figures 3, 5, 6, 7. Lighter circles of the same color denote locations to which the stimulus was subsequently moved during home training. This occurred when performance at the first selected location improved and stabilized (coefficient of variation <20% over at least 5 consecutive days).

Thirteen subacute CB participants [mean ± standard deviation (SD): 7.5 ± 3.8 weeks poststroke] and 12 chronic CB participants (27.6 ± 33.0 months poststroke) took part in this study. Participant demographics, training assignments, and history are detailed in Table 1. Although six chronic participants were naive, three (CB003, CB008, and CB011) trained first as subacutes and then as chronics in the present study. Another set of three chronic participants participated in two prior, unrelated studies, where they learned to discriminate the motion of high-contrast, random dot stimuli (Saionz et al., 2020; Cavanaugh et al., 2023; as detailed in Table 1). All participants sustained stroke-induced, unilateral damage to the occipital lobe in adulthood, causing unilateral, homonymous visual field defects (Fig. 1). Stroke damage was confirmed by brain imaging (Fig. 1), and visual defects were confirmed using 10-2 and 24-2 Humphrey visual fields (HVFs), collected and analyzed as described below. Eligible participants were required to have reliable HVFs (fixation losses, false positives, and false negatives <20%); they had to demonstrate stable and accurate fixation verified by an eye tracker during in-lab psychophysical testing (see below), and they had to be free of ocular or neurological diseases that could interfere with or confound the reason for poor perceptual performance. All participants were best corrected using glasses or contact lenses during testing and training. The Research Subjects Review Board approved study procedures at the University of Rochester, which were conducted as per the Declaration of Helsinki, with written informed consent obtained from each participant, and participation was voluntary.

**Table 1.** Participant demographics and training assignments

Patient code	Sex	Age (years)	Time poststroke	Training tasks Loc. 1, 2	Prior training phase	Prior training task	Pre-post interval (months)	Total training sessions	Compliance Loc. 1, 2 (%)	Deficit area (deg^2^)	Eccentricity Loc. 1, 2 (deg)
Subacute (SA) participants											
CB001	M	47	2.4 wks	Task 1	-	N/A	5.4	40	34	233	21.2
CB002	F	69	6.7 wks	Task 1, 3	-	N/A	4.9	267	126, 128	576	5.8, 5.8
CB003	M	49	6 wks	Task 1, 3	-	N/A	4.6	206	100, 106	586	10.3, 11.2
CB004	M	47	2 wks	Task 1, 3	-	N/A	5.6	217	91, 90	291	14.9, 13.9
CB005	F	27	8.1 wks	Task 1	-	N/A	4.5	19	19	451	10.4
CB006	M	68	3.1 wks	Task 1, 3	-	N/A	5.6	172	66, 75	517	5.8, 10.8
CB007	F	42	5 wks	Task 2, 3	-	N/A	5.4	216	93, 92	509	9.4, 10.3
CB008	M	43	11.4 wks	Task 2, 3	-	N/A	4.0	216	128, 126	753	5.8, 5.8
CB009	M	61	14 wks	Task 1, 2	-	N/A	3.7	206	126, 129	587	11.2, 5.8
CB010	M	68	10.4 wks	Task 1, 2	-	N/A	5.0	233	108, 106	807	5.6, 5.6
CB011	F	61	11.4 wks	Task 1, 2	-	N/A	4.9	241	113, 115	804	5.6, 5.6
CB012	M	60	7.7 wks	Task 2, 3	-	N/A	5.1	188	85, 85	68	5.8, 10.8
CB013	M	68	9.4 wks	Task 2, 3	-	N/A	5.3	301	132, 132	310	9.4, 5.6
Mean ± SD		55 ± 13	7.5 ± 3.8 wks				4.9 ± 0.6	194 ± 80	98 ± 32	500 ± 226	9.2 ± 4.3
Chronic (CH) participants											
CB003	M	50	9.8 mo	Task 1, 3	SA, CH	Task 1, 3, FDD	3.1	149	113, 113	534	11.2,13
CB008	M	44	6.6 mo	Task 1, 2	SA	Task 2, 3	8.2	298	109, 109	740	6.4,5.8
CB011	F	61	7.7 mo	Task 1, 2	SA	Task 1, 2	8.5	311	96, 98	783	6.1,5.6
CB014	M	67	6.9 mo	Task 1, 3	SA	DI	17.8	300	79, 82	65	7.8,10.3
CB015	F	57	42.8 mo	Task 1, 3	CH	DI, FDD	5.9	120	47, 47	447	4.7,8.5
CB017	M	53	33.2 mo	Task 1	CH	FDD	3.5	45	59	377	5.8
CB016	F	63	24.7 mo	Task 1, 3	-	N/A	4.7	244	118, 125	373	8.6,10.8
CB018	M	60	121.4 mo	Task 1, 2	-	N/A	5.1	141	64,64	554	12.1,11.2
CB019	F	73	48.7 mo	Task 2, 3	-	N/A	9.6	277	66, 69	646	5.6,7.8
CB020	F	61	6.3 mo	Task 2, 3	-	N/A	5.8	241	102, 103	576	9.4,11.2
CB021	F	26	7.5 mo	Task 2, 3	-	N/A	5.2	196	86, 89	726	14.9,5.8
CB022	M	72	15.6 mo	Task 1	-	N/A	4.7	88	89	167	6.7
Mean ± SD		57 ± 13	28 ± 33 mo				6.8 ± 4.0	201 ± 91	86 ± 23	499 ± 224	8.6 ± 2.9

Task 1: Static-Ori, orientation discrimination of a static, nonflickering Gabor; Task 2: Flkr-Ori, orientation discrimination of a flickering Gabor; Task 3: Motion, direction discrimination of a drifting Gabor; M, male; F, female; FDD, fine direction discrimination; DI, direction integration; SA, subacute; CH, chronic.

### Perimetric mapping of visual field defects

Monocular 10-2 and 24-2 HVF perimetry was conducted using a Humphrey Field Analyzer II-i750 system (Zeiss Humphrey Systems, Carl Zeiss Meditec) at the University of Rochester Flaum Eye Institute. All tests were performed by the same ophthalmic technicians, with fixation controlled using the system's eye tracker and gaze/blind spot automated controls. Visual acuity was corrected to 20/20 using trial lenses. A white size III stimulus and a background luminance of 11.3 cd/m^2^ were used.

The resulting four test patterns were interpolated using a custom MATLAB (The MathWorks Inc) script to create a unitary, composite HVF map for each participant. Luminance detection thresholds (dB) from monocular HVFs were first averaged between the two eyes and then combined as previously described (Cavanaugh and Huxlin, 2017). The final composite HVF maps (Fig. 1) covered a visual field area of 1,616 deg^2^, stretching a maximum of ±27° along the *x*-axis and ±21° in the *y*-axis. From these maps, we computed the blind-field borders, drawn as a line encompassing regions of the visual field where visual sensitivity was <10 dB (Fig. 1, red lines on HVF maps). This threshold was chosen per the definition of legal blindness provided by the Social Security Administration. Each in-lab psychophysical test location was then drawn as a circle with a radius of either 2 or 2.5° (matching the stimulus size used) onto its corresponding HVF map (Figs. 1, 3) before being categorized by computing the distance in degrees between the blind-field border and each stimulus center (see below for details).

### Apparatus for in-lab psychophysics with eye tracking

Visual discrimination tasks were performed on a Mac Pro computer with stimuli displayed on a CRT monitor (HP 7217A, 48.5 × 31.5 cm screen size, 1,024 × 640 resolution, 120 Hz frame rate, or Dell N993s, 36.5 × 27 cm screen size, 800 × 600 resolution, 120 Hz frame rate). In all cases, monitor luminance was calibrated using a ColorCal II automatic calibration system (Cambridge Research Systems), and the resulting gamma-fit linearized lookup table was implemented in MATLAB. A viewing distance of 42 cm was ensured using a chin/forehead rest. Eye position was monitored binocularly and continuously using an EyeLink 1000 eye tracker (SR Research) with a sampling frequency of 1,000 Hz and accuracy within 0.25°. All tasks and training were conducted using MATLAB and Psychtoolbox (Brainard, 1997; Pelli, 1997).

### Experimental design

#### Stimuli and tasks (Fig. 2)

Each participant had two in-lab visits, during which they were tested with a battery of two-alternative, forced-choice (2AFC) discrimination tasks. The first visit was used to assess baseline performance in the blind and intact fields and to select appropriate training locations in the blind field. Patients then performed several months of at-home training on one of the pre-tested tasks, before returning to the laboratory for a repeat of baseline tests (see Table 1 for testing intervals). This return visit allowed us to measure changes in performance with eye tracker-enforced fixation at initially tested locations.

**Figure 2.**
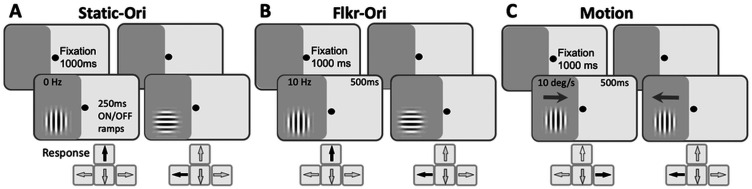
Trial sequences for psychophysical tasks. ***A***, Static-Ori (Task 1): vertical versus horizontal orientation discrimination of nonflickering, static Gabors that appeared over a 250 ms onset, followed by a 250 ms offset. ***B***, Flkr-Ori (Task 2): vertical versus horizontal orientation discrimination of 10 Hz-flickering Gabors with a 500 ms duration. ***C***, Motion (Task 3): left versus right direction discrimination of 10 deg/s drifting Gabors. Participants were asked to first fixate a spot in the middle of the screen for 1,000 ms for stimuli to appear, illustrated here in the impaired hemifield of vision (dark gray shading). Responses were required after each trial. They involved pressing the indicated arrow key on the computer keyboard, followed by auditory feedback as to the correctness of the response.

Stimuli used for in-lab tests and home training were identical. They consisted of Gabor patches (sinusoidal gratings 4–5° in diameter with a Gaussian envelope, sigma 1°) presented on a uniform, mid-gray background, with a 250 ms on/off temporal raised cosine envelope for Task 1 or 500 ms for Tasks 2 and 3. Task 1 (Fig. 2*A*), vertical versus horizontal orientation discrimination of a static, nonflickering Gabor (Static-Ori); Task 2 (Fig. 2*B*), vertical versus horizontal orientation discrimination of a 10 Hz flickering Gabor (Flkr-Ori); Task 3 (Fig. 2*C*), leftward versus rightward direction discrimination of a vertical, drifting Gabor (Motion), whose TF was set to 10 Hz, generating a drift speed of 10 deg/s. SF was set to 1 cycle per degree (cpd) for all tasks. Within each test set of 100 trials, achromatic luminance contrast was titrated using a single two-down one-up staircase with the following steps: 100, 75, 50, 25, 20, 15, 10, 5, 2, and 1%. Contrast thresholds were calculated by fitting a Weibull function to 72.5 percent correct performance, selected because it lies halfway between chance (50 percent correct) and 95 percent correct performance, assuming a 5% lapse rate. CS was calculated as the reciprocal of the contrast threshold.

During in-lab testing, CSFs (specifically, CS vs SF functions) were also measured for all three tasks using a Bayesian, adaptive, quick (qCSF) method (Hou et al., 2010; Lesmes et al., 2010). The CSF indexes the window of visibility. This approach allowed us to estimate contrast thresholds (72.5 percent correct) over a broad SF range in 100 trials (Lesmes et al., 2010). When relevant (i.e., for flickering or drifting Gabors), the TF was set at 10 Hz. For qCSF measurements, stimulus contrast varied between 0.1 and 99% in steps of 1.5 dB, and SF varied from 0.1 to 7.5 cpd over 12 steps (0.10, 0.15, 0.22, 0.32, 0.48, 0.71, 1.05, 1.56, 2.31, 3.42, 5.07, and 7.50 cpd). The stimuli were either 4 or 5° in diameter, and sizing did not scale with SF. The qCSF was expressed as a truncated log-parabola defined by four parameters: peak sensitivity, peak SF, bandwidth at half-height, and low-frequency truncation level. This function allows estimation of the area under the curve (a summary of the entire range of contrast visibility) and the high cutoff SF values (the SF where the contrast threshold is 100%). Participants performed one qCSF run per condition (Static-Ori/Flkr-Ori/Motion), consisting of 100 trials at each visual field location of interest.

#### Mapping and selection of training locations (Fig. 3)

**Figure 3.**
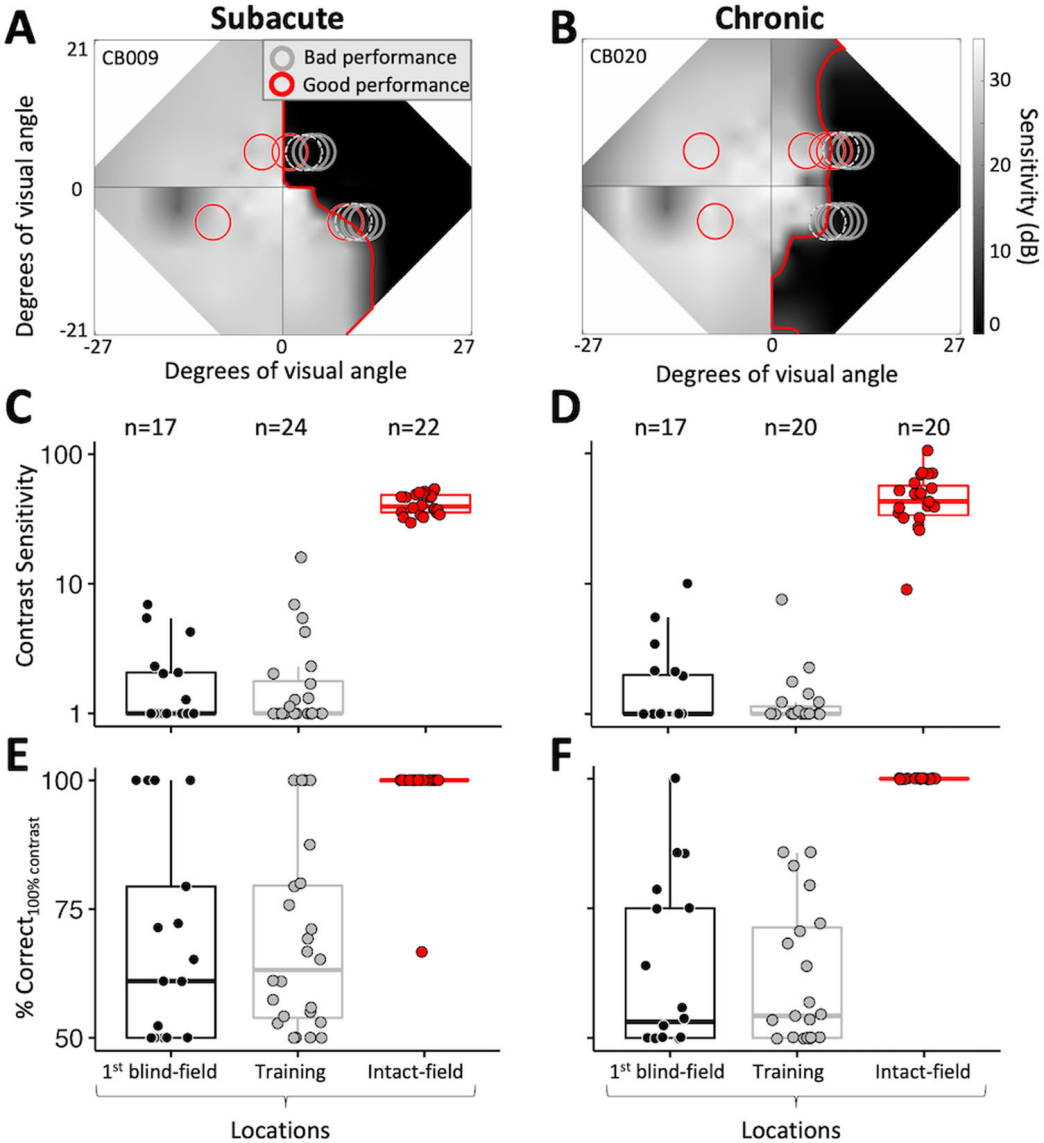
Mapping of baseline performance across the blind-field border. ***A***, Example of locations tested during the mapping process, superimposed on the baseline Humphrey composite map of SA participant CB009. The gray scale denotes luminance detection sensitivity (dB), and the red line denotes the blind-field border. Red circles, locations with Static-Ori performance ≥72.5% correct; gray circles, locations with Static-Ori performance <72.5% correct; dashed gray circles, locations selected for training. Mapping across the border of the blind field moved horizontally from intact to deeper blind-field locations. Once a training location was selected, performance was also measured at mirror-symmetric, intact-field locations (red circles in left hemifield). ***B***, Example of Static-Ori mapping across the blind-field border of a CH participant (CB020). Labeling conventions as in ***A***. ***C***, Plot of CS for Static-Ori in subacute participants at first locations fully inside the blind-field border (1st blind field), selected training locations (Training), and corresponding, intact-field locations. Each data point represents a single location. Box plots indicate the median (line within the box), 25–75% quartile range (box), and 10–90% range (whiskers). Five SA participants had measurable CS (i.e., CS > 1) at seven different first blind-field locations, averaging a CS of 3.5 ± 2.1 at these locations. ***D***, Plot of baseline CS in chronic participants at first locations inside the blind-field border, selected training locations, and corresponding, intact-field locations. Each data point represents a single location. Five CH participants had measurable CS at six different first blind-field locations, with an average CS of 4.2 ± 3.2 at these locations. Labeling convention as in ***C***. ***E***, Percent correct performance on the Static-Ori task for 100% contrast stimuli measured in subacute participants. Four of the 13 subacute participants attained >72.5% correct at seven different first blind-field locations. Labeling conventions as in ***C*** and ***D***. ***F***, Percent correct performance at 100% contrast on the Static-Ori task in chronic participants. Five of the 12 chronic participants attained >72.5% correct at six different first blind-field locations. Labeling conventions as in ***C*** and ***D***.

To characterize visual discrimination performance at different contrasts in each participant, we measured performance on the *Static-Ori* task in the intact visual hemifield across the HVF-defined blind-field border and inside the blind field. Mapping usually started in the intact field or straddling the intact/blind-field border (Fig. 3*A*,*B*), where participants attained relatively normal performance levels (compared with a location of similar eccentricity in their intact hemifield of vision). Each discrimination task began with participants asked to fixate on a centrally presented black spot on the computer monitor in front of them for 1,000 ms. A visual stimulus appeared (see below for description), accompanied by a tone (especially important to signal stimulus appearance in the blind field). The eye tracker monitored eye movements within a 2 × 2° square window centered on the fixation spot. Trials in which eye movements or drifts broke the fixation window were signaled by a loud tone, eliminated from the analysis, and reshuffled into the trial sequence. Participants responded by pressing arrow keys on the computer keyboard, followed by auditory feedback as to the correctness of their response (high-pitched tone, correct response; low-pitched tone, incorrect response). We verified that each participant could hear differences between tones and interpret them correctly. New trials were initiated automatically 500 ms after a response or fixation break.

Participants performed 100 trials of the 1 cpd *Static-Ori* task at each mapping location. Performance ≥72.5 percent correct, with a contrast threshold in the normal range, was “good performance” (Fig. 3*A*,*B*, red circles), and the stimulus was moved laterally along the *x*-axis (Cartesian coordinate space) deeper into the blind field by 1°. Performance was measured at this new location for another 100 trials. The process was repeated until performance fell to a level that prevented a contrast threshold from being computed (i.e., performance <72.5 percent correct). In most participants, several additional locations were also tested deeper in the blind field (Fig. 3*A*,*B*, gray circles) to verify this was a reliable failure point. This mapping procedure allowed us to ascertain how performance changed when stimuli first became fully enclosed within the HVF-defined blind-field border (Fig. 3*A*,*B*, red line) and to select training locations in each participant.

The locations selected for training (Fig. 3*A*,*B*, dotted gray circles) were chosen as the first sites where CS fell below that at corresponding locations in the same person's intact field over a single, 100-trial block after a 1° lateral movement from the intact toward the blind field along the *x*-axis (Cartesian coordinate space). The lowest intact-field CSs for subacutes and chronics were 29.6 and 9, respectively. With few exceptions (e.g., CB014, whose deficit was too small; CB005, whose training program did not work correctly at one location; and CB022, who had preserved motion perception across their entire blind field), participants were trained on two different tasks at nonoverlapping locations along the vertical meridian (Figs. 1, 3*A*,*B*). However, it is important to note that training locations were not always fully enclosed within the perimetrically defined blind-field border; as illustrated by the positioning of some of the dotted gray circles in Figure 3*A*,*B*, participants sometimes exhibited abnormal CS at stimulus locations that partially overlapped the intact field. Nonetheless, 24/46 training locations were located entirely in the blind field, sometimes many degrees deeper than the border. Because of this variance across training locations relative to the border, we compared baseline performance at visual field locations chosen for training and those which first fell entirely within the HVF-defined blind-field border in each participant (Fig. 3*C–F*). Finally, we should also note that while training locations were pre- and post-tested on all three discrimination tasks, each location was trained using only a single task (either Flkr-Ori, Static-Ori, or Motion—see Fig. 1 for color-coded representation of task assignment per location).

#### At-home visual training

After baseline measurements in-lab, participants were sent home to train for several months [subacute (SA), 4.9 ± 0.6 months [mean ± SD]; chronic (CH), 6.8 ± 4.0 months; Table 1]. They used their personal computers with a chin/forehead rest provided by the lab, which they were instructed to position 42 cm away from their display during training. Participants performed 300 trials of their assigned training tasks (Table 1, Fig. 1) per location per day, at least 5 d per week, and they emailed their data log files back to the lab for analysis every week. During home training sessions, they were instructed to stay fixated on the fixation spot and warned that inadequate fixation accuracy could limit recovery.

**Figure 4.**
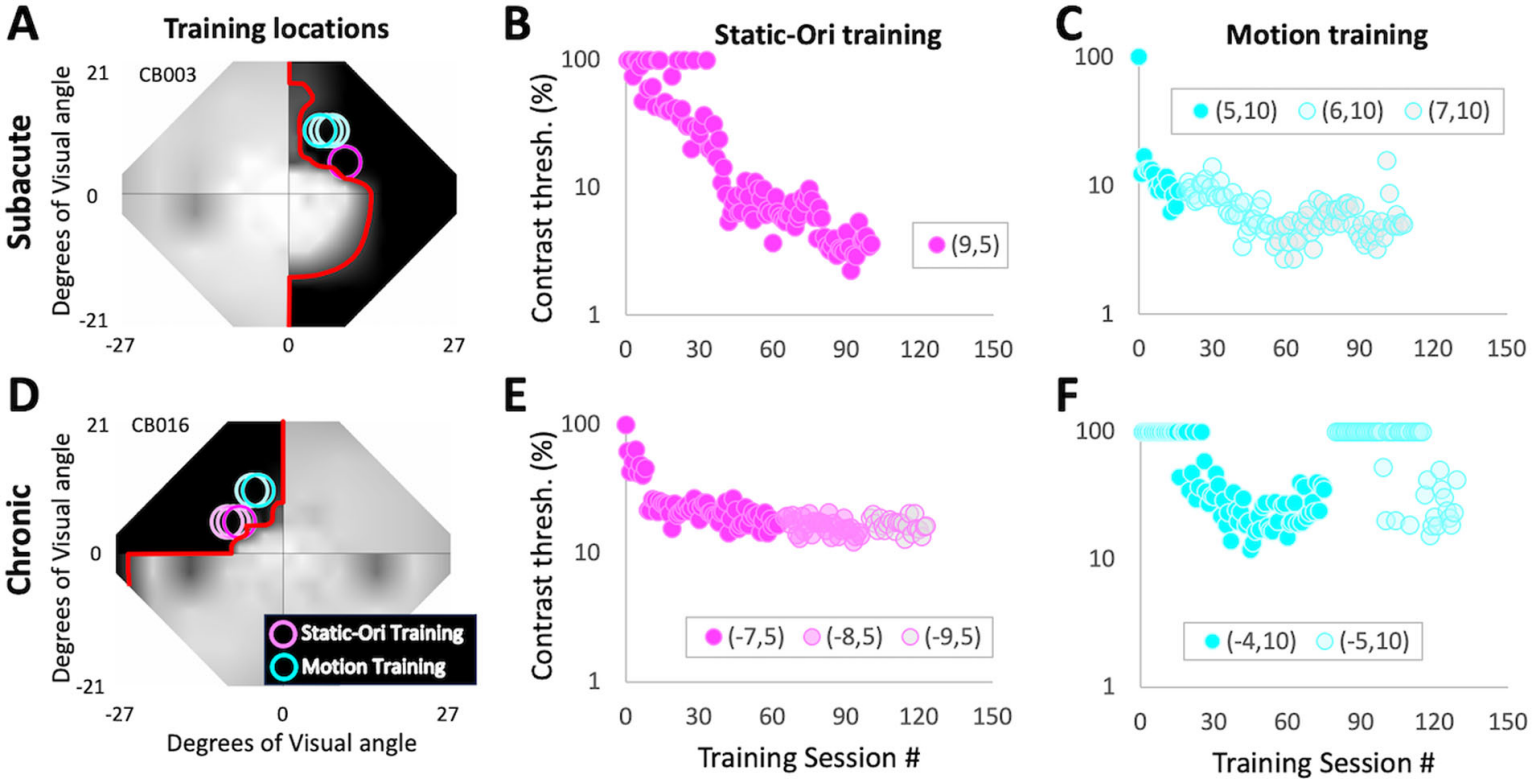
Representative home training performance from one SA and one CH participant. ***A***, Humphrey perimetry-derived, binocular map of visual sensitivity across the central visual field for SA participant CB003 showing locations trained at home on the Static-Ori and Motion tasks. Labeling conventions as in Figure 1. ***B***, CB003's contrast thresholds for each session performed at home on the Static-Ori task. Thresholds dropped gradually before stabilizing. ***C***, Corresponding plot for the same participant's Motion task performance. Here, thresholds dropped relatively fast at the first training location (5,10) and once they stabilized, the training stimulus was moved 1° deeper into the blind field along the *x*-axis (Cartesian coordinate space). Training at the new location—centered on (6,10)—improved further, resulting in the stimulus being moved deeper by 1°, to a new center location of (7,10). ***D***, Humphrey perimetry-derived, binocular map of visual sensitivity across the central visual field for CH participant CB016, showing home training locations. Labeling conventions as in ***A***. ***E***, Home training performance as a function of training session number for CB016's Static-Ori-trained locations. Labeling conventions as in ***B***. ***F***, Home training performance for CB016's Motion-trained locations. Labeling conventions as in ***C***. Both participants had excellent compliance (100% or better) and trained at two different locations daily on two different tasks.

Session thresholds were calculated by fitting a Weibull function with a 72.5 percent correct performance threshold criterion. Once participants’ thresholds improved and stabilized (coefficient of variation <20% over at least 5 consecutive days) at the first training location, their training stimulus was moved 1° deeper into the blind field along the *x*-axis (Cartesian coordinate space). Because stimulus radius was 2–2.5°, all new training locations had ∼80% overlap with the prior location (see Figs. 1, 4 for illustration). Once participants trained for ∼4 months, with at least one improved location (defined as consistently good contrast thresholds at that location), they were brought back to the lab, and performance at all home-trained locations was verified with eye tracker-enforced fixation control. We aimed for a similar number of training sessions at the blind-field locations of interest before scheduling people to return for in-lab performance verification and calculated the total number of sessions trained across all training locations per participant (Table 1, Fig. 1). However, the amount of time elapsed until the return visit did vary, as it was affected by the individual's rate of improvement, their work/family schedules, and ability to travel to our single study site (participants originated from across the entire United States and Canada, and some were caught in COVID pandemic lockdowns, rendering them unable to return to lab at their appointed time). For this reason, we also computed participant compliance (Table 1), defined as the number of training sessions performed at each blind-field location as a percentage of the number of prescribed training sessions [one training session per location per day multiplied by the number of prescribed training days (5 d/week) between the last day of the pre-training visit and the first day of the post-training visit]. The home training performance data for each participant and each task can be found on FigShare (10.6084/m9.figshare.24739581).

### Statistical analyses

Training locations were treated as independent due to the nonuniform nature of hemianopic visual field defects, both in terms of baseline discrimination performance and training-induced changes in performance (Huxlin et al., 2009; Das et al., 2014; Saionz et al., 2020). Although two locations were sampled in each person's blind field, only one intact-field location was tested in some participants due to time constraints or participant exhaustion. For this reason, Mann–Whitney (two groups) or Kruskal–Wallis tests with post hoc Dunn's multiple-comparisons tests (more than two groups) were used. *H* statistics are provided for 1 (*H*_1_) or 2 (*H*_2_) degrees of freedom.

Because of the adaptive nature of the qCSF procedure, a bootstrap method was used to determine statistically significant changes across individuals. Individual trials were first randomly resampled 2,000 times with replacement to generate a resampled trial sequence, which was refitted using the qCSF procedure. From these 2,000 qCSFs at each test location, we repeated the resampling procedure 10,000 times to generate bootstrap distributions of the means of the fitted parameters and the CS means at each SF. We used a peak CS <2.55 as our chance level performance, and thus any trial with peak sensitivity <2.55 was set to zero (for detailed methods, see Saionz et al., 2020). We used a three-way analysis of variance (ANOVA) for unpaired samples between pre-training and intact-field CSFs. To compute *p* values for comparisons between pre- and post-training CSFs, we used paired Student’s *t* tests to compare the means of each parameter and CS means at each SF. For group comparisons at each of the 12 SF levels, *p* values were Bonferroni corrected for multiple comparisons.

## Results

### Baseline CS for static orientation discrimination at 1 cpd

In their intact hemifield of vision, SA participants, who were all naive to training, averaged (±SD) 75.2 ± 1.3 percent correct overall and a CS of 41.7 ± 7.4 on the Static-Ori task, whereas CH participants performed comparably, averaging 75 ± 2 percent correct with a CS of 48.5 ± 21.5. Individual data for both groups, along with medians and quartiles are shown in Fig. 3*C* (SA) and 3*D* (CH). Eccentricities tested ranged from 5.6 to 21.2° for SA and from 5.6 to 14.9° for CH participants (Table 1), with no effect of eccentricity on CS (SA: *r*_20_ = −0.25, *p* = 0.26; CH: *r*_18 _= −0.21, *p* = 0.38).

At selected training locations, baseline performance on the Static-Ori task was markedly lower in both SA (61 ± 9 percent correct; CS = 2.3 ± 3.3; Fig. 3*C*) and CH participants (60 ± 9 percent correct; CS = 1.5 ± 1.5; Fig. 3*D*). As with the intact-field locations, eccentricity did not appear to influence CS in either patient group (SA: *r*_22 _= −0.06, *p* = 0.78; CH: *r*_18 _= 0.08, *p* = 0.74). At first locations inside the perimetrically defined blind-field border, SA (Fig. 3*C*) and CH (Fig. 3*D*) participants’ average CSs were 2.0 ± 1.8 and 2.1 ± 2.4, respectively. Less than half of participants [SA (5/13), CH (5/12)] had partial preservation of CS (>1), with most of them able to correctly discriminate static orientation at >72.5 percent correct when stimuli were 100% contrast (Fig. 3*E*,*F*).

There was a significant main effect of test location on CS (Kruskal–Wallis; *H*_2 _= 86.2, *p* < 0.001), percent correct (*H*_2 _= 79.2, *p* < 0.001), and percent correct at 100% contrast (*H*_2 _= 66.6, *p* < 0.001). A post hoc Dunn's test for multiple-comparisons using a Bonferroni-adjusted alpha level of 0.017 (0.05/3) showed the main effect of test location to be driven by intact-field locations, where performances were significantly better than at training locations and first locations fully in the blind field. However, there was no significant effect of participant type on performance [CS (*H*_1 _= 0.03, *p* = 0.87), percent correct (*H*_1 _= 0.01, *p* = 0.92), percent correct at 100% contrast (*H*_1 _= 1.07, *p* = 0.3)]. In sum, the baseline performance of SA and CH participants was comparably good in their intact fields and impaired at first locations fully inside the blind-field border, which were as impaired as locations selected for training. As such, SA and CH participants were well matched in terms of performance prior to the onset of training. From here on, we will only detail performance changes for selected training locations.

Finally, we should also note that for CH participants, there was no significant impact of prior training on CS (Kruskal–Wallis; *H*_1 _= 2, *p* = 0.16) and overall percent correct (*H*_1 _= 0.16, *p* = 0.69) at all locations tested at baseline. Naive and previously trained CH participants had similar baseline performance at selected training locations and at first locations fully inside their blind field.

### Baseline CS for discrimination of flickering and moving Gabors

**Figure 5.**
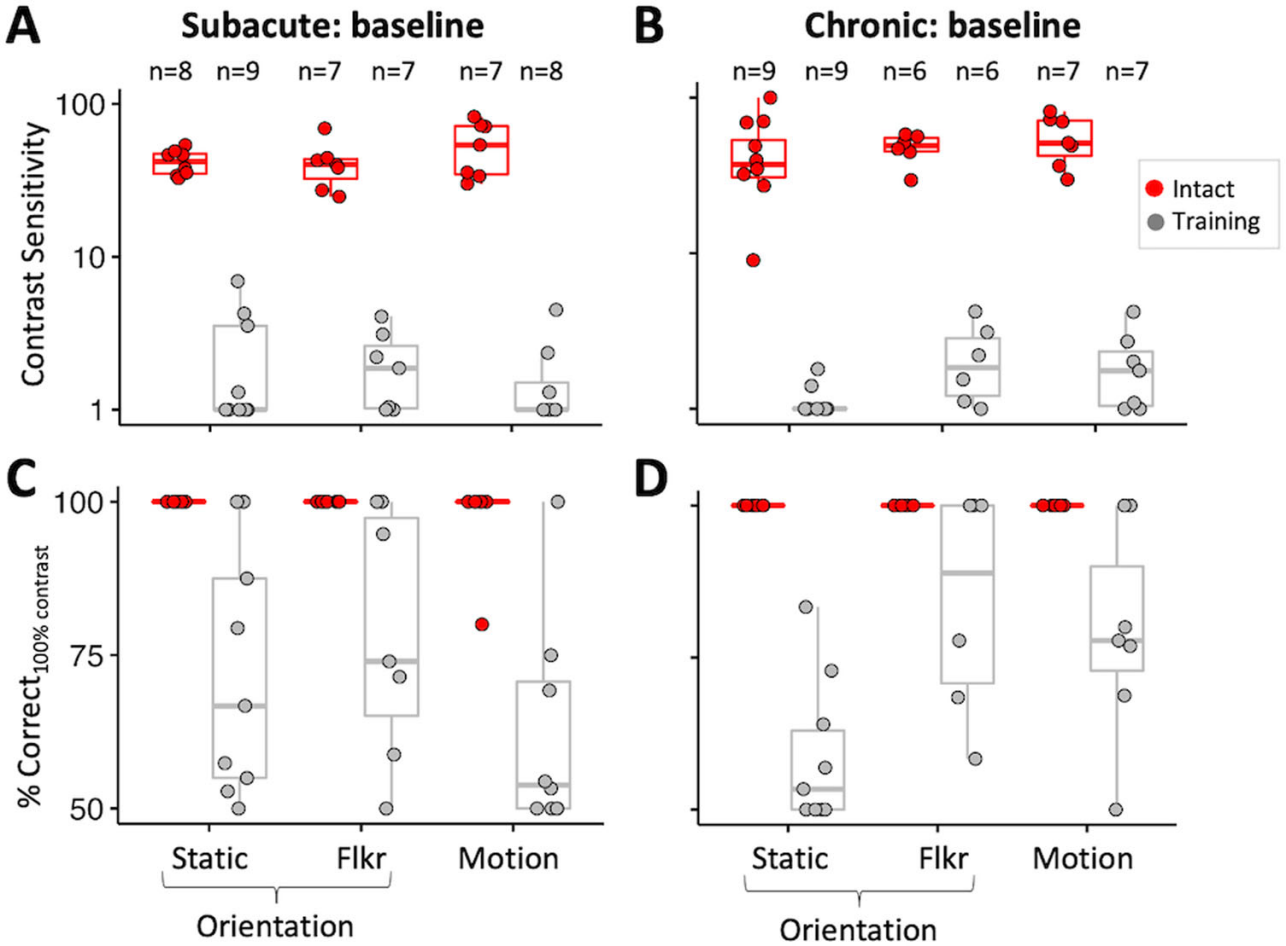
Baseline performance on all three discrimination tasks. ***A***, Plot of CS for orientation discrimination of static Gabors (Static), flickering Gabors (Flkr), and direction discrimination of drifting Gabors (Motion) in subacute participants at selected training locations and corresponding, intact-field locations. Each data point represents a single location. Red-filled circles indicate performance at corresponding intact-field locations; gray-filled circles denote locations selected for training. Box plots indicate the median (line within the box), 25–75% quartile range (box), and 10–90% range (whiskers). ***B***, Plot of CS for the same three tasks in chronic participants at locations selected for training and their corresponding intact-field locations. Labeling conventions as in ***A***. ***C***, Baseline percent correct performance on three tasks for 100% contrast stimuli measured in subacute participants. Labeling conventions as in ***A*** and ***B***. ***D***, Baseline percent correct performance on three tasks for 100% contrast stimuli measured in chronic participants. Labeling conventions as in ***A–C***.

Key questions in the present study were whether the addition of temporal modulation or motion signals would improve baseline CS in the blind field of people with V1 damage. Thus, we remeasured baseline performance after adding a 10 Hz flicker to the orientation discrimination task (Flkr-Ori) or changing the task to a direction discrimination (Motion) at locations selected for training on the basis of static orientation discrimination. Kruskal–Wallis tests of data in Figure 5 showed no significant main effects of participant group or task on baseline CS (group: *H*_1 _= 0.05, *p* = 0.82; task: *H*_2 _= 3.91, *p* = 0.14), overall percent correct (group: *H*_1 _= 0.41, *p* = 0.52; task: *H*_2 _= 5.12, *p* = 0.08), or percent correct at 100% contrast (group: *H*_1 _= 0.08, *p* = 0.77; task: *H*_2 _= 4.86, *p* = 0.09). Thus, there was no benefit of adding 10 Hz TF modulation or motion information at blind-field locations selected for training on the basis of static orientation discrimination in either SA (Fig. 5*A*,*C*) or CH (Fig. 5*B*,*D*) participants.

### Effect of training on the trained tasks

#### SA participants

**Figure 6.**
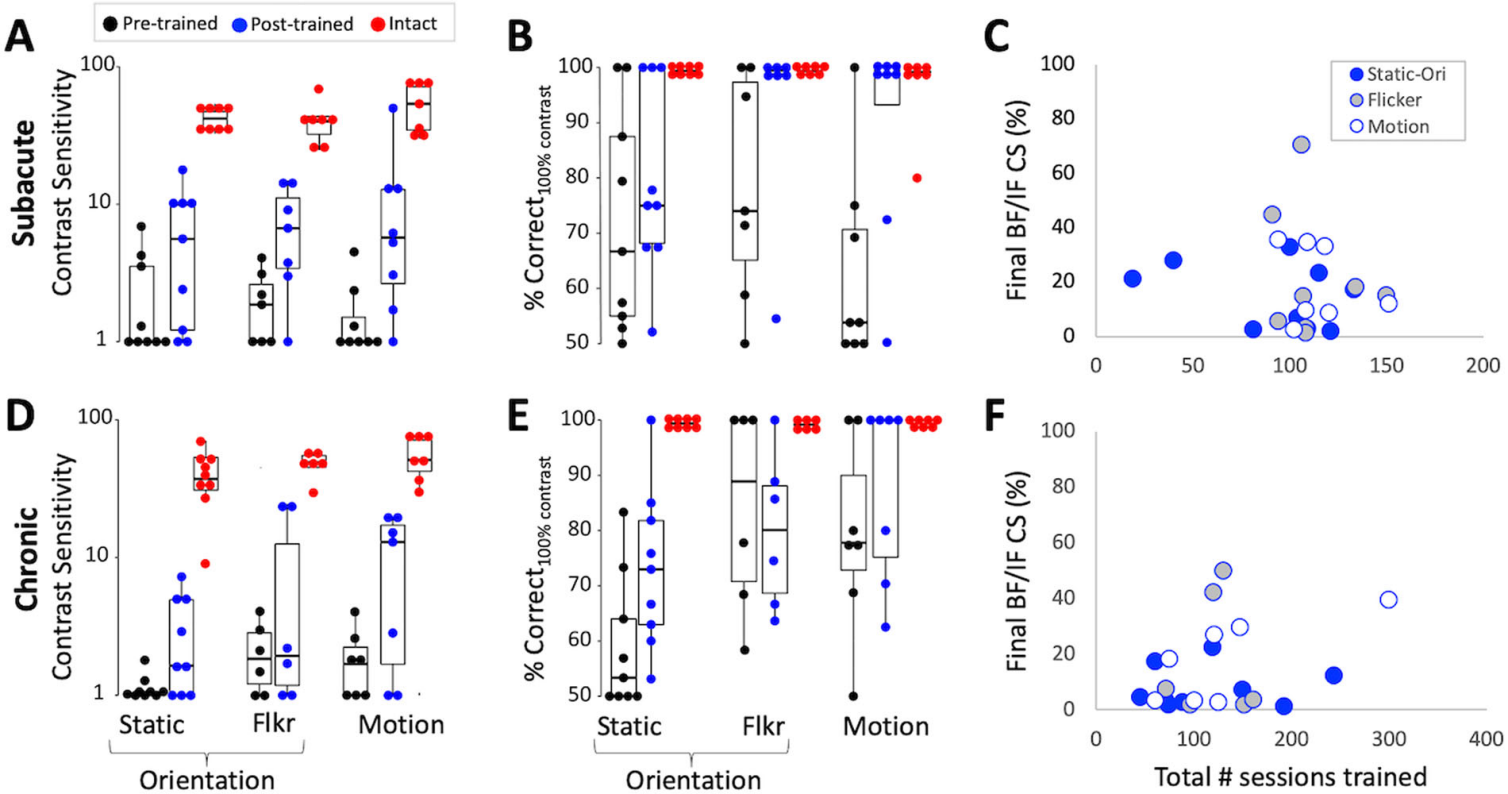
Effects of training on trained discrimination tasks in SA and CH participants. ***A***, Pre- (black dots) and post-training (blue dots) CS for all three tasks at locations initially selected for training. Intact-field performance is shown for reference (red dots). Each data point represents a single location. Box plots show the median (line within the box), 25–75% quartile range (box), and 10–90% range (whiskers) at each time point. ***B***, Pre- and post-training percent correct performance at 100% contrast for all three tasks, along with performance in the intact field. Labeling conventions as in ***A***. ***C***, Final CS attained by each SA participant expressed as a percentage of each person's intact-field CS, plotted against the total number of sessions trained. None of the patients recovered normal CS and there was no significant correlation between the amount of CS regained and the number of sessions trained. ***D***, Pre- and post-training CS for all three tasks, along with performance in the intact field in CH participants. Labeling conventions as in ***A***. ***E***, Pre- and post-training percent correct performance at 100% contrast for all three tasks, along with performance in the intact field of the same CH participants in ***D***. Labeling conventions as in ***A***. ***F***, Plot of the final CS attained by each CH participant as a percentage of each person's intact-field CS versus the total number of sessions trained. None of the CH participants recovered CS in the blind field that was comparable to that in their intact field and there was no significant correlation between the amount of CS regained and the number of sessions trained.

Direction/orientation discrimination training improved CS by at least 1 unit in 12/13 SA participants (92%) at 17/24 training locations (71%). Five participants improved at both of their trained locations, 7 at 1 trained location, and 1 at neither trained location. Across tasks, CS improved at 6/9 Static-Ori–trained locations (67%; Fig. 6*A*), 5/7 Flkr-Ori–trained locations (71%; Fig. 6*A*), and 5/8 Motion-trained locations (62.5%; Fig. 6*A*). Overall, post-training CS (mean 8.5 ± 10.2 for three tasks) was significantly higher than pre-training CS (2.0 ± 1.6; Wilcoxon signed-rank; *V* = 205, *p* = 0.0002), although there were no significant differences in CS improvements among training tasks (Kruskal–Wallis; *H*_2 _= 0.56, *p* = 0.76). However, post-training CS across all training tasks remained significantly impaired compared with corresponding intact-field locations (45.5 ± 15.8; Wilcoxon rank sum; *W* = 16, *p* < 0.001; Fig. 6*A*). There was also a significant overall improvement in percent correct performance for 100% stimulus contrast (87 ± 18%; Wilcoxon signed-rank; *V* = 183.5, *p* = 0.004), again, without significant differences among training tasks (*H*_2 _= 2.87, *p* = 0.24; Fig. 6*B*). Post-training overall percent correct performance (Wilcoxon rank sum; *W* = 95, *p* = 0.0002) and percent correct performance for 100% stimulus contrast (*W* = 172.5, *p* = 0.006) also remained significantly impaired compared with intact-field locations.

Even when the analysis was restricted to locations that improved, the average CS increase across tasks was 9.8 ± 11.3, with no significant effect of the training task (Kruskal–Wallis; *H*_2 _= 1.02, *p* = 0.6). Mean post-training CS was 12 ± 11, which was approximately six times better than baseline (2.2 ± 1.8; Wilcoxon signed-rank; *V* = 136, *p* < 0.001) but remained approximately four times lower than mean CS in the same participants’ intact fields (45.2 ± 16.1; Wilcoxon rank sum; *W* = 12, *p* < 0.001).

#### CH participants

Training improved CS by at least 1 unit in fewer people (7/12, 58%) and at fewer trained locations (10/22, 45%) than that in SA participants. Of the seven CH participants whose CS improved, three did so at both trained locations, and four at one location. Across tasks, improvement was seen at 4/9 (44%) locations for the Static-Ori task (Fig. 6*C*), 2/6 (33%) locations for the Flkr-Ori task (Fig. 6*C*), and 4/7 (57%) locations for the Motion task (Fig. 6*C*). Overall, post-training CS (6.9 ± 8) was significantly higher than at baseline (1.7 ± 1; Wilcoxon signed-rank; *V* = 146, *p* = 0.001), with no significant differences in CS improvements among training tasks (Kruskal–Wallis; *H*_2 _= 0.67, *p* = 0.72). Unlike SA participants, there was no systematic improvement across all CH participants and conditions in percent correct performance for 100% contrast stimuli (Wilcoxon signed-rank; *V* = 145, *p* = 0.14; Fig. 6*D*). As a result, mean post-training CS (Wilcoxon rank sum; *W* = 6, *p* < 0.001; Fig. 6*C*), percent correct (*W* = 58.5, *p* < 0.001), and percent correct for 100% stimulus contrast (*W* = 66, *p* < 0.001) all remained significantly impaired in CH participants compared with their intact-field locations.

When restricting the analysis to the 10 impaired locations that improved in CH participants, the average increase in CS was 11.3 ± 7.2, but the training task had a significant effect (Kruskal–Wallis, *H*_2 _= 7.9, *p* = 0.02). A post hoc Dunn's test for multiple-comparisons using a Bonferroni’s-adjusted alpha level of 0.017 (0.05/3) showed this effect to be driven by a smaller CS change at Static-Ori–trained locations (3.8 ± 1.6) than at Flkr-Ori– and Motion-trained locations. Finally, mean post-training CS at improved locations across all three tasks reached 13.4 ± 8, approximately six times better than the baseline (2.1 ± 1.3; Wilcoxon signed-rank; *V* = 55, *p* = 0.002), but once again, approximately four times less than CS in the same participants’ intact fields (59.2 ± 21; Wilcoxon rank sum; *W* = 0, *p* < 0.001).

In summary, most SA participants showed CS improvement following training, and these improvements were similar across tasks. Conversely, only half of CH participants had improved CS, and for them, adding TF content generated greater improvements. However, in all cases (SA and CH), post-training CS remained significantly impaired relative to that in the intact fields (Fig. 6). Even when restricting the analysis to blind-field locations that benefited from training, CS remained ∼4–5 times lower than that in the intact fields, with no significant differences in CS improvements between participant type (SA or CH; Kruskal–Wallis; *H*_1 _= 0.8, *p* = 0.37). However, the training task had a significant effect on overall CS changes at improved locations (*H*_2 _= 6.58, *p* = 0.04). A post hoc Dunn's test for multiple comparisons showed this effect to be driven by a smaller CS change at Static-Ori–trained locations (5.6 ± 4.4) than at Motion-trained locations (15.2 ± 13.6). Only one SA Motion-trained participant attained a CS of 50 (in Fig. 6*A*), which fell within the intact-field range for this task (CS, 30–82).

### Effect of training amount and compliance on CS change

On average, SA participants trained for 49 ± 36, 34 ± 27, and 53 ± 37 sessions of 300 trials each at Static-Ori–, Flkr-Ori–, and Motion-trained locations, respectively, equating to compliance (or training density) of 87 ± 40%, 112 ± 18%, and 104 ± 22%. CH participants trained for 59 ± 44, 60 ± 24, and 43 ± 22 sessions at Static-Ori–, Flkr-Ori–, and Motion-trained locations, equating to compliance of 86 ± 25%, 88 ± 19%, and 90 ± 27%, respectively. Thus, compliance was excellent in the present study, and there was no significant effect of participant group (Wilcoxon rank sum; *W* = 355.5, *p* = 0.05) or training task (Kruskal–Wallis; *H*_2 _= 1.31, *p* = 0.52) on compliance. Importantly, we found no significant correlations between compliance (SA: *r* = −0.09, *p* = 0.55; CH: *r* = −0.04, *p* = 0.79) or number of training sessions (SA: *r* = −0.14, *p* = 0.33; CH: *r* = −0.16, *p* = 0.34) and changes in CS attained by either SA or CH participants. Thus, it is unlikely that more training would have further improved performance.

### Transfer of learning to untrained SFs

**Figure 7.**
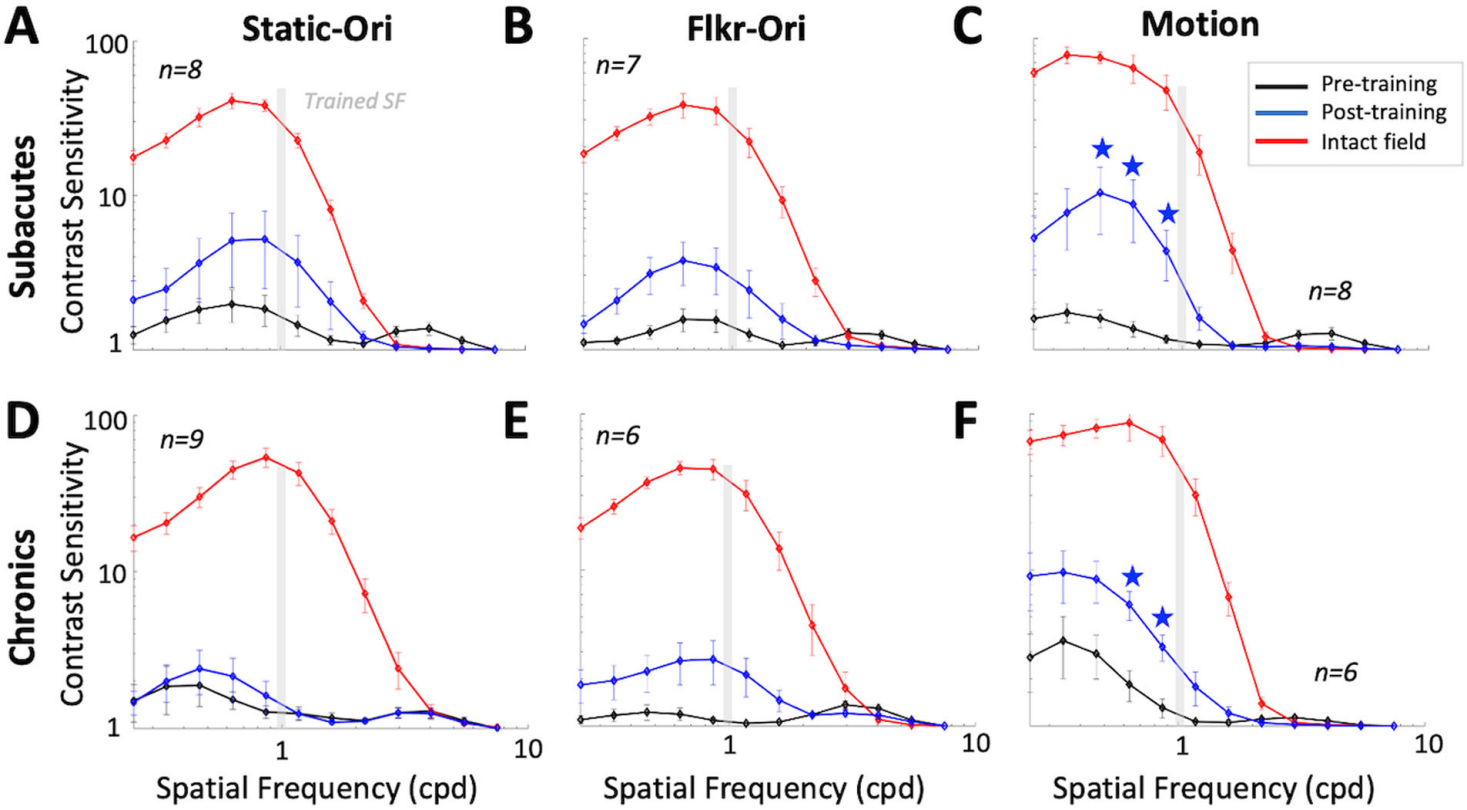
Effect of training on CSFs for Static-Ori, Flkr-Ori, and Motion tasks in SA and CH participants. ***A***, Mean pre- (black line) and post-training (blue line) CSFs for Static-Ori task in SA participants, at locations selected for training as well as corresponding, intact-field locations (red line). ***B***, Corresponding CSFs for Flkr-Ori task in SA participants. Labeling conventions as in ***A***. ***C***, Corresponding CSFs for Motion task in SA participants. Labeling conventions as in ***A***. ***D***, Corresponding CSFs for Static-Ori task in CH participants. Labeling conventions as in ***A***. ***E***, Corresponding CSFs for Flkr-Ori task in CH participants. Labeling conventions as in ***A***. ***F***, Corresponding CSFs for Motion task in CH participants. Labeling conventions as in ***A***. Error bars, SEM. Light gray vertical bars, trained SF (1 cpd). Within each graph, average CSs at each of the 12 SFs tested (0.10, 0.15, 0.22, 0.32, 0.48, 0.71, 1.05, 1.56, 2.31, 3.42, 5.07, 7.50 cpd) were corrected for multiple comparisons using Bonferroni’s correction, followed by bootstrapping analysis at each SF, with blue stars denoting *p* < 0.004.

The qCSF method estimates the CS versus SF function as a truncated log-parabola with four parameters. Prior to training, a three-way ANOVA [variables: location (intact, blind), task (Static-Ori, Flkr-Ori, Motion), and patient type (SA, CH); Fig. 7*A–F*] confirmed severely impaired qCSFs at locations selected for training versus intact-field locations, whether in terms of peak SF (*F*_(1,77) _= 12.64, *p* = 0.0006), bandwidth at half-height (*F*_(1,77) _= 32.12, *p* < 0.001), and low-frequency truncation level (*F*_(1,77) _= 99.29, *p* < 0.001). Unsurprisingly, differences extended to mean area-under-the-curve (AUC; pre-training loc, 1.5 ± 1.4; intact loc, 72.6 ± 2.6; *F*_(1,77) _= 594.33, *p* < 0.001) and mean peak CS (pre-training loc, 2.3 ± 1.7; intact loc, 58.8 ± 1.7; *F*_(1,77) _= 923.67, *p* < 0.001). There was a significant interaction between location and task (*F*_(2,77) _= 4.42, *p* = 0.02) on peak CS; this was driven by higher intact-field peak CS for motion (99.5 ± 1.6) than Flkr-Ori (45.1 ± 1.5, *p* = 0.002) and Static-Ori (48.1 ± 1.6, *p* = 0.002), together with a lack of such differences at locations selected for training. Motion qCSFs also had a lower mean low-frequency truncation level (reflecting better sensitivity at low SFs; 0.2 ± 1.4) and narrower mean bandwidth (1.7 ± 1.3) at intact-field locations compared with Flkr-Ori and Static-Ori qCSFs and a smaller mean peak SF (0.8 ± 1.5) compared with Static-Ori qCSFs at intact-field locations. Importantly, these results showed no significant effect of the participant group (SA vs CH) on baseline qCSFs in terms of the abovementioned parameters. In sum, qCSFs were comparable in SA and CH participants at baseline, with no consistent benefit of adding TF modulation or motion information at blind-field locations selected for training in either group.

Bootstrap analysis (Fig. 7) revealed a significant change in the qCSFs only for SA and CH participants trained on the Motion task (Fig. 7*C*,*F*). Both SA (5.7 ± 3.1 post- vs 2.3 ± 1.4 pre-training; two-tailed paired Student's *t* test; *t*_(7) _= −2.53, *p* = 0.04; Fig. 7*C*) and CH participants (8.4 ± 2.6 post- vs 2.6 ± 2.8 pre-training; *t*_(5) _= −8.2, *p* = 0.0004; Fig. 7*F*) improved the mean peak CS at Motion-trained locations. Similarly, the mean area under the Motion qCSFs was approximately two times that of pre-training for both SA (3.5 ± 2.8 post- vs 1.5 ± 1.3 pre-training; *t*_(7) _= −2.82, *p* = 0.03) and CH (4.2 ± 2.1 post- vs 1.7 ± 1.8 pre-training; *t*_(5) _= −4.68, *p* = 0.005) participants.

Although training was at 1 cpd, improved CS for Motion transferred to lower SFs (0.2–0.5 cpd; Fig. 7*C*,*F*, stars) for both SA and CH participants, which was reflected in a reduced, post-training, low-SF truncation level (SA: *t*_(7) _= 3, *p* = 0.02; CH: *t*_5 _= 3.39, *p* = 0.02). However, for both SA and CH participants, post-training AUC and peak CS amplitude at Motion-trained locations remained significantly impaired compared with those at intact-field locations (SA: AUC *t*_(12)_ = −5.00, *p* = 0.0003; peak ampl. *t*_(8)_ = −6.81, *p* = 0.0001; CH: AUC *t*_(5)_ = −4.85, *p* = 0.005; peak ampl. *t*_(5)_ = −4.79, *p* = 0.005).

## Discussion

The present study examined CS after stroke-induced V1 damage and asked to what extent this basic property of vision can be restored inside the blind field, whose boundaries were defined using a new, principled approach. Although orientation or motion discrimination was sometimes preserved, CS was severely impaired from the earliest time poststroke, irrespective of whether stimuli were static, flickering, or moving. Our results show—for the first time—that the best-known approach to improve perception (training targeted at the deficient function, bootstrapped to tasks known to elicit improvements in CB patients) fails to restore normal CS when V1 is damaged. The perceptual training employed here has never been attempted in CB, let alone in a side-by-side comparison of subacute and chronic stroke patients. Saionz et al. (2020) contrasted training effects between subacute and chronic CB patients, but this was done using a high-contrast, global direction discrimination task with random dot stimuli—not contrast-varying, oriented Gabors—as the goal in this prior study was to improve global direction discrimination and integration. Our results are also novel in the context of understanding what a primate visual system with a damaged V1 is capable of relearning and what it is not. Specifically, we now show an inability of V1-damaged humans to recover CS back to normal, using a task that would typically improve CS in visually intact participants. This inability stands in stark contrast to the same patients’ ability to regain normal direction integration (Huxlin et al., 2009; Saionz et al., 2020), direction discrimination (Cavanaugh et al., 2019), and orientation discrimination (Das et al., 2014) in their blind field, with high-contrast stimuli. As such, this is the first report of a sensory ability that cannot be restored in V1-damaged patients despite using the best training protocols at our disposal. As detailed below, this finding indicates possibly fundamental limitations for recovery of this visual attribute in V1-damaged humans.

### V1 damage impairs CS, even when orientation and direction discrimination are preserved

All CB participants had pronounced deficits in peripheral CS for discriminating Gabors inside, as well as straddling, their perimetrically defined blind-field border, confirming prior reports with less precise definitions of the blind field (Das et al., 2014; Saionz et al., 2020) and those using detection rather than discrimination tasks (Hess and Pointer, 1989; Barbur et al., 1994; Sahraie et al., 2008; Ajina et al., 2015; Ajina and Bridge, 2019). Importantly, our earliest patients (∼2 weeks poststroke) and chronic participants were similarly affected. This suggests a severe, early deficit in CS for discrimination after V1 stroke in spite of normal discrimination of high-contrast stimuli for some patients and normal CS at intact-field locations in all patients.

There are mixed reports of preserved conscious visual abilities in perimetrically defined blind fields after V1 damage. For detection, some (Blythe et al., 1987; Barbur et al., 1994; Sahraie et al., 2008; Ajina et al., 2015; Ajina and Bridge, 2019), but not others (Hess and Pointer, 1989), reported preservation. For discrimination, most failed to find preservation for direction (Azzopardi and Cowey, 2001; Huxlin et al., 2009; Mazzi et al., 2016; Saionz et al., 2020) or orientation (Morland et al., 1996; Das et al., 2014; Mazzi et al., 2016; Saionz et al., 2020). However, to this point, definitions of the blind-field border have varied across studies. Here, using a new, principled method for defining this border, we found that approximately half our stroke participants could reliably describe stimuli and perform discrimination tasks at contrast-impaired locations when luminance contrast was 100%. Thus, computations for discriminating large orientation and direction differences inside perimetrically defined blind fields may be distinct (and longer-lasting poststroke) than those required for CS. For visually intact humans, CS for discriminating and detecting low-SF motion stimuli is similar, suggesting that these processes are mediated by the same, directionally selective mechanisms (Watson et al., 1980; Watson and Robson, 1981; Anderson and Burr, 1991). However, strobe-reared cats, whose early visual neurons fail to develop direction selectivity, display reduced CS for discrimination, but not detection (Pasternak et al., 1985; Pasternak and Leinen, 1986; Pasternak and Tadin, 2020). They can only discriminate direction at high contrasts (Pasternak and Leinen, 1986). Cats with lesions of area 17, the homolog of primate V1, also display greater deficits for discrimination than detection of grating orientation and motion direction (Pasternak et al., 1995). Thus, in V1-damaged humans, residual orientation- and direction-selective units across the residual visual system may support the ability of some individuals to discriminate large orientation or direction differences at high contrast in their blind fields. However, the loss of a large number of orientation- and direction-selective neurons in V1 means that CS for discriminating these features is severely diminished, even when detection persists.

### Better training efficacy in subacute than chronic stroke participants

Over 90% of subacute but only half of chronic participants exhibited training-induced improvements in CS, despite comparable compliance and number of sessions trained. Additionally, subacutes improved comparably across tasks, whereas chronics improved less on the Static-Orientation task. Why should time since stroke make such a difference, especially for static stimuli? A potential factor is progressive, trans-synaptic retrograde degeneration of early visual pathways initiated by the V1 lesion (Beatty et al., 1982; Cowey et al., 1989, 2011; Jindahra et al., 2009, 2012; Bridge et al., 2011; Park et al., 2013; Herro and Lam, 2015; Fahrenthold et al., 2021). Retrograde degeneration is thought to primarily affect parvocellular neurons in the dorsal lateral geniculate nucleus and retina (Cowey et al., 1989)— cells most sensitive to stationary stimuli, high SF and low TF content (Derrington and Fuchst, 1979; Foster et al., 1985; Sclar et al., 1990). Subacute participants exhibit fewer signs of retrograde degeneration than chronic participants (Fahrenthold et al., 2021), supporting the notion that better integrity of early visual pathways could underlie their better contrast-training outcomes.

### Neural mechanisms underlying limited CS restoration in CB

When developing therapeutic tools, it is equally important to define functions that can and cannot be restored and understand why. Although adaptive-contrast training improves CS for direction and orientation discrimination in visually intact humans (Levi et al., 2015; Xi et al., 2020), we now report its failure to restore normal CS in V1 stroke patients. Instead, it improved CS similarly to training with high-contrast stimuli (Huxlin et al., 2009; Das et al., 2014; Saionz et al., 2020). Given that CS may be strongly related to the activity (Niemeyer and Paradiso, 2017) and number of V1 neurons (Himmelberg et al., 2022; Jigo et al., 2023) representing particular visual field regions, our findings suggest a potentially fundamental limitation of the V1-damaged visual system: with insufficient V1 neurons, the residual circuitry may simply be incapable of the processing necessary to restore normal CS over affected parts of the visual field. Thus, in CB participants, the amount of spared V1 representing portions of the blind field (Papanikolaou et al., 2014; Barbot et al., 2021), along with maintenance of its retinal and subcortical input, may be vital to maximize recovery of CS.

### Partial transfer of CS learning to lower SFs

A central question in perceptual training is whether learning transfers to untrained features. Here, we asked if training with 1 cpd contrast-varying Gabors altered the qCSF at untrained SFs. The qCSFs measured in the intact fields of CB patients were comparable to those measured in young, visually intact controls (Levi et al., 2015), as well as in the intact field of CB patients (Das et al., 2014). Importantly, here we used similar stimulus parameters (size, SF and TF, eccentricity) and discrimination tasks as in those two studies. Training with high-contrast, random dot stimuli improved CS for discriminating low-SF drifting gratings in both CB (Huxlin et al., 2009; Das et al., 2014; Saionz et al., 2020) and visually intact (Levi et al., 2015) humans. Similarly, we saw significant changes in qCSF at low SFs (0.6–1 cpd) for motion discrimination in both subacute and chronic participants. Thus, training with motion stimuli appears to generate improvements in CS, which transfer to lower SFs. After V1 damage, this may be consistent with a greater, relative contribution of spared, motion-sensitive extrastriate areas such as MT/V5, to CS; MT neurons are broadly tuned but prefer lower SFs (Pawar et al., 2019). In nonhuman primates, they remain responsive, even months after V1 damage, although direction selectivity decreases over time (Rodman et al., 1989; Girard et al., 1992; Rosa et al., 2000; Azzopardi et al., 2003; Hagan et al., 2020).

### Methodological developments

This study presents a new, principled method for registering behavioral performance measured in-lab with Humphrey perimetry—the most common clinical test for assessing vision loss in CB. On the Humphrey test, when patients cannot see a stimulus 10,000 apostilbs in light intensity, that location is assigned a value of 0 dB. Here, we drew the blind-field border as linking sites where sensitivity fell below 10 dB on the Humphrey, consistent with the US Social Security Administration definition of blindness. Surprisingly, not only could some CB participants discriminate orientation of high-contrast stimuli fully inside this blind-field border, sometimes, contrast thresholds were severely impaired at locations that straddled the border and included large regions of “intact” vision. We propose that the discrepancy between Humphrey perimetry and in-lab CS measures is rooted in differences between stimuli, tasks, and threshold strategies. Humphrey perimetry displays small (0.43°), broadband (in SF content), white lights on a bright background (10 cd/m^2^) for ∼200 ms each, whereas our CS tasks used larger, 1 cpd Gabors on a brighter background (120 cd/m^2^) for 500 ms. Humphrey perimetry also randomly measures detection at equally spaced test locations across the central ±21–27° of vision, whereas our discrimination task mapped performance at very few, adjacent, overlapping sites, using systematic, 1° lateral displacements of the stimulus after each set of 100 trials. The differences in visual performance resulting from such simple changes in low-level features of stimuli and tasks suggest that great care is needed when defining “blindness” in the context of V1 strokes.

### Conclusions

CS defines thresholds for visibility and discrimination—both fundamental properties of human vision. V1 strokes in humans provide us with a unique “experiment of nature”—a natural intervention that allowed us to test predictions from the literature about the critical role of V1 in the perception of luminance contrast, in addition to answering novel questions about whether the residual visual circuitry has the potential to recover CS in the context of discrimination tasks and whether intervention time matters. We now report that CS in the visual hemifield contralateral to an occipital stroke degrades rapidly, whereas orientation and motion discrimination of high-contrast stimuli can persist in a proportion of participants for several months. Adaptive-contrast training improved CS in the blind field but still failed to restore it to normal levels. However, more subacute than chronic stroke participants benefitted from such training, particularly when discriminating the orientation of static targets. Our results support the notion that CS may be critically dependent on processing within V1 and indicate that intervention time matters. As such, maximizing recovery of CS within cortically blinded fields may require early intervention and/or preserving the integrity of early visual pathways.

## Synthesis

Reviewing Editor: Matthew Grubb, King's College London Decisions are customarily a result of the Reviewing Editor and the peer reviewers coming together and discussing their recommendations until a consensus is reached. When revisions are invited, a fact-based synthesis statement explaining their decision and outlining what is needed to prepare a revision will be listed below. The following reviewer(s) agreed to reveal their identity: Aaron Seitz. Note: If this manuscript was transferred from JNeurosci and a decision was made to accept the manuscript without peer review, a brief statement to this effect will instead be what is listed below.

Reviewer 1:

The authors have addressed all the concerns in my review for the Journal of Neuroscience.

Reviewer 2:

The manuscript is significantly improved and I endorse publication
